# Investigation of H_2_S Donor Treatment on Neutrophil Extracellular Traps in Experimental Colitis

**DOI:** 10.3390/ijms222312729

**Published:** 2021-11-25

**Authors:** Szilvia Török, Nikoletta Almási, Zsuzsanna Valkusz, Anikó Pósa, Csaba Varga, Krisztina Kupai

**Affiliations:** 1 Department of Physiology, Anatomy and Neuroscience, Faculty of Science and Informatics, University of Szeged, H-6726 Szeged, Hungary; tszilvia@bio.u-szeged.hu (S.T.); almasi@expbio.bio.u-szeged.hu (N.A.); paniko@bio.u-szeged.hu (A.P.); vacs@bio.u-szeged.hu (C.V.); 2 Albert Szent-Györgyi Clinical Center, Department of Medicine, Medical Faculty, University of Szeged, H-6720 Szeged, Hungary; valkusz.zsuzsanna@med.u-szeged.hu; 3 Interdisciplinary Excellence Center, Department of Physiology, Anatomy and Neuroscience, Faculty of Science and Informatics, University of Szeged, H-6726 Szeged, Hungary

**Keywords:** hydrogen sulfide, inflammation, inflammatory bowel disease, colitis, neutrophil extracellular traps

## Abstract

Inflammatory bowel diseases (IBD) are chronic, immune-mediated disorders, which affect the gastrointestinal tract with intermittent ulceration. It is increasingly clear that neutrophil extracellular traps (NETs) seem to have a role in IBD; however, the associated pathogenesis is still not known. Furthermore, several conventional therapies are available against IBD, although these might have side effects. Our current study aimed to investigate the effects of hydrogen sulfide (H_2_S) treatment on NETs formation and on the expression of inflammatory mediators in experimental rat colitis. To model IBD, 2,4,6-trinitrobenzenesulfonic acid (TNBS) was administered intracolonically (i.c.) to Wistar–Harlan male rats. Animals were treated (2 times/day) with H_2_S donor Lawesson’s reagent per os. Our results showed that H_2_S treatment significantly decreased the extent of colonic lesions. Furthermore, the expression of members of NETs formation: peptidyl arginine deiminase 4 (PAD4), citrullinated histone H3 (citH3), myeloperoxidase (MPO) and inflammatory regulators, such as nuclear transcription factor-kappa B (NF-κB) and high-mobility group box 1 (HMGB1) were reduced in H_2_S treated group compared to TNBS. Additionally, H_2_S donor administration elevated the expression of ubiquitin C-terminal hydroxylase L1 (UCHL-1), a potential anti-inflammatory mediator. Taken together, our results showed that H_2_S may exert anti-inflammatory effect through the inhibition of NETs formation, which suggests a new therapeutic approach against IBD.

## 1. Introduction

Inflammatory bowel diseases (IBD) are chronic, immune-mediated disorders, which affect the gastrointestinal (GI) tract with intermittent ulceration. Despite extensive research, the pathogenesis of IBD is not precisely known. IBD occurred worldwide at the turn of the 21st century with stabilized incidence but still high prevalence in industrialized countries and with a continuous increase in developing countries [1]. Crohn’s disease (CD) and ulcerative colitis (UC) are the two major types of IBD with similar symptoms but different epidemiological and clinical features [2]. Both disorders are characterized by phases of relapses and remissions, weight loss, fever, abdominal pain, diarrhea, and rectal bleeding [3]. These symptoms affect the quality of life of the patients, so the primary purposes of IBD therapy are the induction and maintenance of remission along with symptomatic treatments [4]. Currently, several therapeutic options exist [5], although they often cause side effects [6]. Therefore, the development of new therapies and potential therapeutic targets are urgently needed.

Several animal models have been established to investigate IBD, which represent the symptomatic, morphological, and histopathological features of human IBD. Among them, chemically induced IBD models, such as 2,4,6-trinitrobenzenesulfonic acid (TNBS), dextran sodium sulfate (DSS), oxazolone, or acetic acid colitis models are widely used because of their cost-effectiveness, good reproducibility, and relative simplicity of induction [7]. The TNBS-induced colitis model was originally described by Morris et al. [8]. In rodents, a single intracolonic administration of the hapten reagent (TNBS) dissolved in ethanol results in serious transmural inflammation and ulceration of the colon. As a vehicle, ethanol causes destruction of the mucosal barrier, which enables TNBS to access proteins in the inner layers of the colonic tissue. Interaction of the hapten with colonic proteins initiates Th-1-mediated immune response [9]. Based on the aforementioned features, TNBS-induced colitis model is a suitable tool for investigating the pathogenesis of IBD and developing novel therapeutic options against these ailments.

Hydrogen sulfide (H_2_S) was known only as a toxic gas for many years, but in the last two decades, it has emerged as an important mediator of normal physiological pathways in various biological systems [10]. H_2_S is generated endogenously in mammals by many enzymatic and non-enzymatic pathways [11,12]. Several investigations revealed that H_2_S has a wide range of regulatory functions and plays a role in many physiological and pathological processes [13,14,15,16]. In the GI tract, H_2_S plays a role in normal smooth muscle function [17], mucosal defence [18], and in the stimulation of epithelial secretion [19]. In pathological conditions, H_2_S seems to reduce inflammation by the enhancement of ulcer healing [20,21].

Neutrophil infiltration is a typical phenomenon in IBD [22]. Neutrophils play a central role in innate immunity, as they are the first immune cells that migrate to the site of inflammation [23]. In response to infection or inflammatory stimuli, neutrophils can form neutrophil extracellular traps (NETs), a web-like structure, which consists of DNA, histone proteins, granular proteins, such as neutrophil elastase (NE) and myeloperoxidase (MPO), and cytoplasmatic components [24,25]. During NETs formation, ROS-induced peptidyl arginine deiminase 4 (PAD4) activation forms citrullinated histone H3 (citH3), which initiates chromatin decondensation [26]. Then NETs are released into the extracellular environment to immobilize and catch pathogens [27,28]. The process of producing and releasing NETs is referred to as NETosis, which was described by Brinkmann et al. in 2004 [24]. Although NETs play an important role in host defence by protecting from infections, excessive formation of NETs is involved in the pathogenesis of several inflammatory diseases and tissue damage [27,28,29]. Accordingly, members of NETs formation can be used as important biomarkers in several ailments [28,30] and for potential therapeutic targets [31,32,33]. Recently, few studies suggested the relationship between H_2_S and NETs formation [34,35].

Besides various stimuli, such as pathogens, cytokines and enhanced ROS production, high-mobility group box 1 (HMGB1), a proinflammatory mediator, can also induce NETs formation via Toll-like receptor 4 (TLR4) [36]. Additionally, it is demonstrated that the levels of fecal HMGB1 correlates with the severity of mucosal inflammation; therefore, HMGB1 is suggested as a fecal biomarker of IBD [37]. NF-κB, as a principal regulator of inflammatory processes, is involved in the pathogenesis of several inflammatory diseases, including IBD [38]. Several animal models of inflammatory diseases demonstrated that the obstruction of the NF-κB signalling pathway is an effective treatment by inhibiting inflammation and tissue damage [38,39,40]. Furthermore, it is increasingly clear that ubiquitin C-terminal hydroxylase L1 (UCHL-1), a deubiquitinating enzyme, may have a role in the reduction of inflammation [41,42]. In addition, in vascular cells, it was shown that UCHL-1 suppressed NF-κB activation via deubiquitinating IκB-α, therefore preventing its proteasomal degradation [43].

Therefore, in this current study, we hypothesized that H_2_S can inhibit the colonic NETs formation in a rat model of colitis and may contribute to the reduction of inflammation and the healing of ulcers. Our results showed that an exogenously administered H_2_S donor suppressed the expression of the members of NETs formation and other inflammatory mediators in TNBS-induced colitis. Furthermore, H_2_S donor treatment increased the levels of UCHL-1, a potential candidate for anti-inflammatory processes. Based on our results, we suggest that NETs components may serve as potential therapeutic targets in IBD and H_2_S administration seems to provide a promising strategy against IBD through the inhibition of NETosis.

## 2. Materials and Methods

### 2.1. Experimental Animals

All procedures were performed in accordance with the standards of the European Community guidelines for the Care and Use of Laboratory Animals and were approved by the Institutional Ethics Committee (XX./4799/2015, 15 December 2015) at the University of Szeged.

Male Wistar–Harlan rats (225–250 g) were purchased from Toxi-Coop Ltd. (Budapest, Hungary) and were kept in a conventional animal house under controlled conditions (temperature 22 ± 2 °C; relative humidity 55 ± 10%), with a 12 h light-dark cycle. Standard rat chow and tap water were supplied ad libitum throughout the experiments.

### 2.2. Drug Preparations

To induce colitis 2,4,6-trinitrobenzenesulfonic acid (TNBS) was prepared in 50% ethanol (EtOH) and distilled water mixture. Lawesson’s reagent suspended in 0.5% carboxymethylcellulose (CMC) was used as an H_2_S donor. As a positive control, sulfasalazine (SASP) was applied, which was dissolved in physiological saline (0.9%). All chemicals were purchased from Sigma-Aldrich (St. Louis, MO, USA) and were prepared freshly before treatments. Animals were anesthetized with thiopental (Tiobarbital Braun, 0.5 g, B. Braun Medical SA, Barcelona, Spain) dissolved in physiological saline (0.9%).

### 2.3. Experimental Design and Drug Administration

After one week of acclimatization rats were divided randomly into three groups: absolute control (no treatment, *n* = 8), EtOH group (0.25 mL 50% ethanol enema, *n* = 8), and TNBS group (10 mg in 0.25 mL of 50% ethanol, *n* = 34). The experimental colitis was achieved based on Morris’ method [8]. Following overnight fasting, animals were challenged with a single dose of 0.25 mL TNBS intracolonically (i.c.) with an 8 cm long soft polyethylene tube through the anus under mild anesthesia (thiopental, i.p. 40 mg/kg). Next, the TNBS group was divided further and treated immediately after TNBS instillation per os for 3 days (twice/day) with the following drugs: Lawesson’s reagent as a H_2_S donor (TNBS+ H_2_S donor, *n* = 16) at dose of 18.75 µM/kg/day (dissolved in 0.5% CMC, *n* = 8); SASP as a positive control (TNBS+SASP, *n* = 10) at dose of 50 mg/kg/day (dissolved in physiological saline (0.9%); CMC (0.5%, vehicle of Lawesson’s reagent). The selection of the drug doses was based on our previous findings in the same experimental colitis model [44]. Rats were euthanized (thiopental, i.p. 100 mg/kg) 72 h after TNBS instillation and the distal 8 cm portion of the colon was excised, longitudinally opened, washed with ice-cold physiological saline, and photographed (Panasonic Lumix DMC-TZ6, Panasonic Co., Kadoma, Osaka, Japan) for determination of macroscopic inflammation. Finally, the colon samples were frozen and powdered in liquid nitrogen and kept at −80 °C until used for biochemical analysis.

### 2.4. Evaluation of Macroscopic Inflammatory Damage

The extents of macroscopically apparent inflammation, ulceration, and tissue necrosis were determined in a randomized manner from the colored images, using proprietary computerized planimetry software developed in our laboratory (Stat_2_1_1, Szeged, Hungary), based on planimetrics. The area of macroscopically visible mucosal lesion was calculated and represented as the percentage (%) of the total 8 cm colonic segment area.

### 2.5. Measurement of Colonic MPO Enzyme Activity

The colonic inflammatory damage was also determined by the measurement of myeloperoxidase (MPO) activity, employing a modified method described by Bradley et al. [45]. MPO is primarily expressed in neutrophil granulocytes, and the activity of the enzyme is commonly used to quantify neutrophil infiltration. Approximately 30 mg of frozen, powdered colon tissue samples were homogenized (Benchmark Scientific Handheld homogenizer D1000, 13,500 rev/min, 2 × 10 s, Benchmark Scientific, NJ, MA, USA) in ice-cold phosphate buffer (50 mM, pH 6.0), containing 0.5% hexadecyltrimethylammonium-bromide (HETAB). To further break cell membranes, homogenates were subjected to freeze-thaw cycles four times in liquid nitrogen and a 37 °C water bath. Then, homogenates were centrifuged at 10,000× *g* for 15 min at 4 °C. In a 96-well plate, 12 µL from each supernatant was mixed with 280 µL phosphate buffer (50 mM, pH 6.0) containing 0.167 mg/mL *O*-dianisidine dihydrochloride (Sigma-Aldrich, St. Louis, MO, USA), and the reaction was initiated with 10 μL of 0.03% hydrogen peroxide. After shaking for 90 s, the activity of MPO was detected spectrophotometrically at 490 nm (Benchmark Microplate Reader, Bio-Rad Laboratories, Hercules, CA, USA). Results were expressed as µU/mg protein.

### 2.6. Expression of the Inflammatory Mediators and NETosis Markers by Western Blot Analyses

In order to detect expression changes of inflammatory mediators and NETosis markers Western blot analyses were performed. Approximately 30 mg of frozen, powdered colon samples were homogenized with an ultrasonicator (UP100H Hielscher, 3 × 10 s, Teltow, Germany) in Radioimmunoprecipitation assay (RIPA) buffer (Merck Millipore, Burlington, MA, USA), supplemented with phenylmethanesulfonyl fluoride (PMSF) (Sigma-Aldrich, St. Louis, MO, USA) with a tissue to homogenization buffer ratio of 1:4. The supernatants were collected by centrifugation at 12,000 rpm for 10 min at 4 °C. After determination of protein concentrations by Bradford assay, 50 µg protein of each lysate was mixed with reducing sample buffer (Thermo Scientific, Waltham, MA, USA), heated to 95 °C for 5 min and loaded onto 10% sodium dodecyl sulfate (SDS)-polyacrylamide gels. After separation by electrophoresis (90 V, 2 h), proteins were transferred onto nitrocellulose membranes (Amersham Pharmacia Biotech., Buckinghamshire, UK) for 2.5 h at 35 V. The transfer efficiency was checked by Ponceau staining. Membranes were blocked with 5% non-fat dry milk, except for citH3 (5% bovine serum albumin (BSA)), in Tris-buffered saline containing 0.05% Tween 20 (TBS-T) overnight at 4 °C. After three times 10 min washing in TBS-T, membranes were incubated with primary antibodies in 1% milk or in case of citH3 in 1% BSA, for 2 h at room temperature (RT) or overnight at 4 °C: anti-PAD4 (1:1000, overnight, Proteintech, Rosemont, IL, USA, 17373-1-AP), anti-citH3 (1:750, 2 h, Abcam, Cambridge, UK, ab5103), anti-MPO (1:1000, overnight, Abcam, Cambridge, UK, ab208670), anti-HMGB1 (1:1000, 2 h, Abcam, Cambridge, UK, ab79823), anti-NF-κB p65 (1:500, 2 h, Abcam, Cambridge, UK, ab16502), anti-UCHL-1 (1:500, 2 h, Abcam, Cambridge, UK, ab108986). Then membranes were rinsed again for 3 × 10 min in TBS-T and were incubated with anti-rabbit or anti-mouse horseradish peroxidase (HRP)-conjugated secondary antibody (1:5000, DAKO Agilent, Santa Clara, CA, USA) in 1% non-fat dry milk or 1% BSA for 1 h at RT. After washing in TBS-T, membranes were developed with Immobilon Western Chemiluminescent HRP Substrate (Merck Millipore, Burlington, MA, USA) to visualize proteins. Signals were detected by Uvitec gel documentation system (Uvitec Ltd., Cambridge, UK), and densities were analysed by Quantity One Software version 4.5 (Bio-Rad Laboratories, Hercules, CA, USA). Finally, to check the exact amount of the loaded proteins, membranes were stripped, blocked in 5% BSA, and probed with anti-β-actin (1:10,000, Abcam, Cambridge, UK, ab20272) primary antibody in 1% BSA for 2 h at RT, followed by incubation with anti-mouse HRP-conjugated secondary antibody (1:5000; 1 h, RT, DAKO Agilent, Santa Clara, CA, USA), and detected as described above. Results are presented as relative expressions normalized to β-actin.

### 2.7. Protein Determination

The total protein contents were measured by Bradford assay. Aliquots of 20 μL of the diluted samples (25× or 40× with distilled water) were mixed with 980 μL distilled water, and 200 μL of Bradford reagent was added to each sample. Following 10 min of incubation, samples were assayed spectrophotometrically at 595 nm and compared to bovine serum albumin (BSA) standards. Protein concentration was expressed as mg protein/mL.

### 2.8. Statistical Analysis

All data are expressed as mean ± standard error of the mean (S.E.M.). Statistical analysis was carried out by SigmaPlot 12.0 for Windows (Systat Software Inc., San Jose, CA, USA). Parameters were shown normal distribution, and data were assessed by one-way ANOVA followed by Holm–Sidak post hoc test in all measurements. A probability (p) value of less than 0.05 was accepted as statistically significant.

## 3. Results

### 3.1. Protective Effect of Hydrogen Sulfide Donor on the Severity of Inflammation in TNBS-Induced Rat Colitis

**Figure 1:**
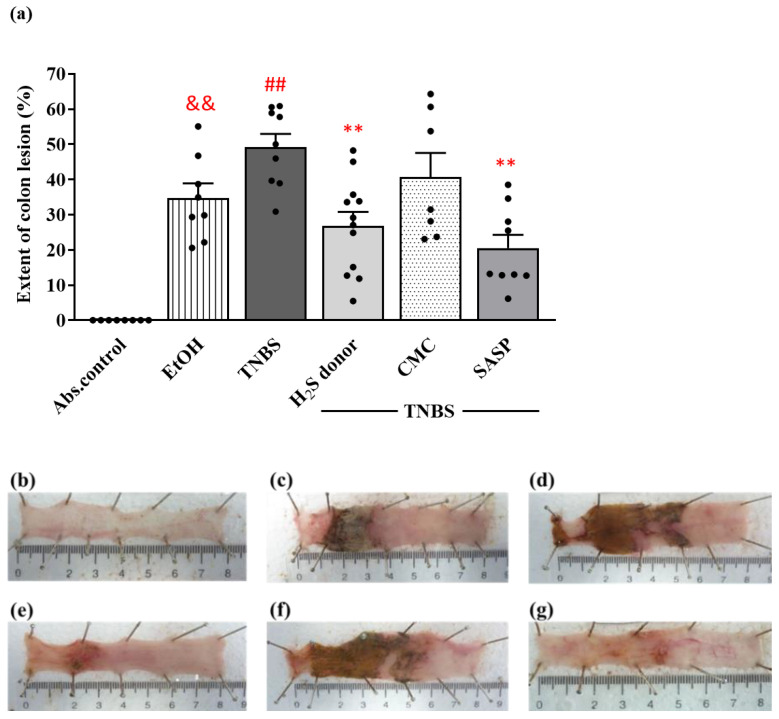
(**a**) The extent of colonic lesion in the different treatment groups in TNBS-induced rat colitis. Representative images of colonic inflammation in the experimental groups: (**b**) absolute control (no treatment); (**c**) EtOH (50% ethanol, the solvent of TNBS); (**d**) TNBS (2,4,6-trinitrobenzenesulfonic acid enema); (**e**) H_2_S donor (TNBS + 18.75 µM/kg/day Lawesson’s reagent); (**f**) CMC (TNBS + 0.5% carboxymethylcellulose, the vehicle of Lawesson’s reagent); (**g**) SASP (TNBS + 50 mg/kg/day sulfasalazine, as a positive control). Results are shown as mean + S.E.M.; *n* = 7–12/group; one-way ANOVA, Holm–Sidak post hoc test, && *p* < 0.001 absolute control vs. EtOH; ## *p* < 0.001 absolute control vs. TNBS; ** *p* < 0.001 TNBS vs. TNBS + treatments.

To model colitis, 2,4,6-trinitrobenzenesulfonic acid (TNBS) dissolved in 50% ethanol (EtOH) was given intracolonically to rats, and 72 h after challenge, the macroscopic colonic injuries were analysed. Both TNBS and its solvent EtOH provoked serious colonic mucosal damage compared to the untreated healthy controls. EtOH, as the vehicle of TNBS, caused hyperaemia and superficial ulcers of the colonic tissue. However, the administration of TNBS resulted in a more intense injury, as approximately 50% of the examined colonic area were damaged with inflammation and obvious hemorrhagic necrosis. In our previous experiment, we determined the most effective dose of H_2_S donor Lawesson’s reagent (18.75 µM/kg/day), and as a positive control sulfasalazine (SASP) (50 mg/kg/day), which both decreased the severity of the colonic inflammation [44]. Our present study confirmed that treatment with an 18.75 µM/kg/day dose of H_2_S donor significantly reduced the extent of colon lesions compared to the TNBS group (26.89 ± 2.92 vs. 49.28 ± 3.71%). Similarly, a statistically significant attenuation was found in the severity of inflammation in the positive control group, treated with SASP (20.49 ± 3.63 vs. 49.28 ± 3.71%). CMC, the vehicle of Lawesson’s reagent, was also tested alone and showed no significant difference in the colonic damage compared to the TNBS group, thus suggesting that CMC has no effect on inflammation. The extent of the macroscopically apparent mucosal injury was expressed as the percentage of the total colonic segmented area (Figure 1a), and representative images of the colon are shown in Figure 1b–g.

### 3.2. Oral administration of Hydrogen Sulfide Donor Reduced the Elevated Expressions of PAD4 NETosis Marker by TNBS

**Figure 2:**
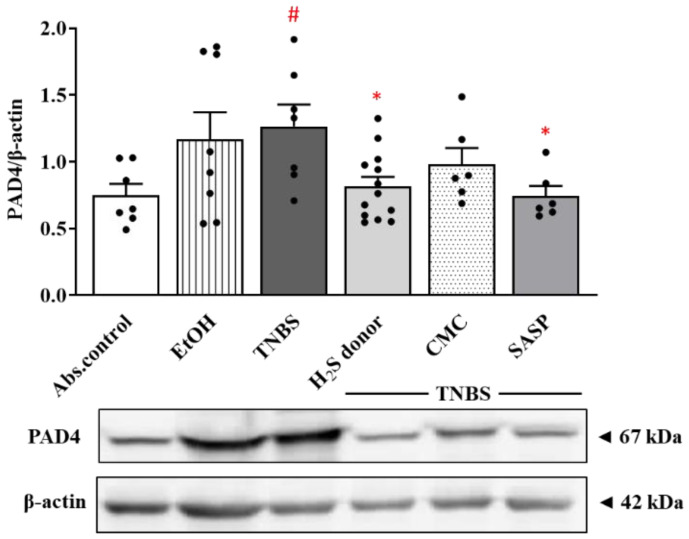
Changes of the expression of PAD4 NETosis marker after H_2_S donor administration. Absolute control (no treatment); EtOH (50% ethanol, the solvent of TNBS); TNBS (2,4,6-trinitrobenzenesulfonic acid enema); H_2_S donor (TNBS + 18.75 µM/kg/day Lawesson’s reagent); CMC (TNBS + 0.5% carboxymethylcellulose, the vehicle of Lawesson’s reagent); SASP (TNBS + 50 mg/kg/day sulfasalazine, as a positive control). Results are presented as mean + S.E.M.; *n* = 6–13/group; one-way ANOVA, Holm–Sidak post hoc test, # *p* < 0.05 absolute control vs. TNBS; * *p* < 0.05 TNBS vs. TNBS + treatments.

In this study, the expression of the NETosis marker PAD4 was determined by western blotting (Figure 2). PAD4 showed a statistically significant elevation after TNBS administration compared to the control group (1.26 ± 0.16 vs. 0.75 ± 0.08 relative expression). The expression of PAD4 was significantly reduced by H_2_S donor and SASP treatment compared to the TNBS group (0.82 ± 0.07 and 0.74 ± 0.07 vs. 1.26 ± 0.16 relative expression).

### 3.3. Alteration in the Expression of Colonic citH3 following Treatments

**Figure 3:**
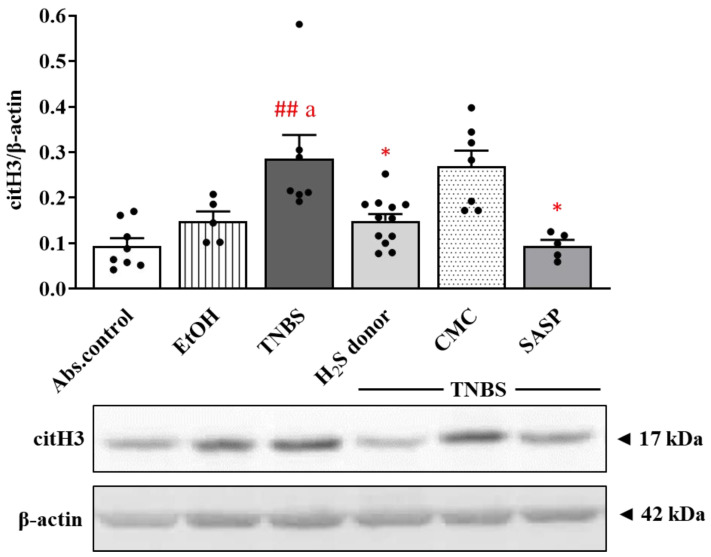
The expression of citH3 after TNBS administration and H_2_S donor treatments in rat colon tissues. Absolute control (no treatment); EtOH (50% ethanol, the solvent of TNBS); TNBS (2,4,6-trinitrobenzenesulfonic acid enema); H_2_S donor (TNBS + 18.75 µM/kg/day Lawesson’s reagent); CMC (TNBS + 0.5% carboxymethylcellulose, the vehicle of Lawesson’s reagent); SASP (TNBS + 50 mg/kg/day sulfasalazine, as a positive control). Results are presented as mean + S.E.M.; *n* = 5–12/group; one-way ANOVA, Holm–Sidak post hoc test, ## *p* < 0.001 absolute control vs. TNBS; a *p* < 0.05 EtOH vs. TNBS; * *p* < 0.05 TNBS vs. TNBS + treatments.

PAD4-induced citrullination of histone H3 is also used as a marker of NET formation. Corresponding to the results of PAD4, we observed similar trends in the case of citH3 (Figure 3). The expression of the citrullinated protein showed a significant elevation in TNBS-induced inflammation in the colon compared to absolute control and EtOH groups (0.29 ± 0.05 vs. 0.09 ± 0.018 and 0.15 ± 0.02 relative expression); however, both H_2_S donor and SASP administration significantly decreased the level of citH3 relative to the TNBS group (0.15 ± 0.02 and 0.09 ± 0.01 vs. 0.29 ± 0.05 relative expression).

### 3.4. Hydrogen Sulfide Donor Treatment Suppressed the Activity and Expression of MPO in Rat Colon

**Figure 4:**
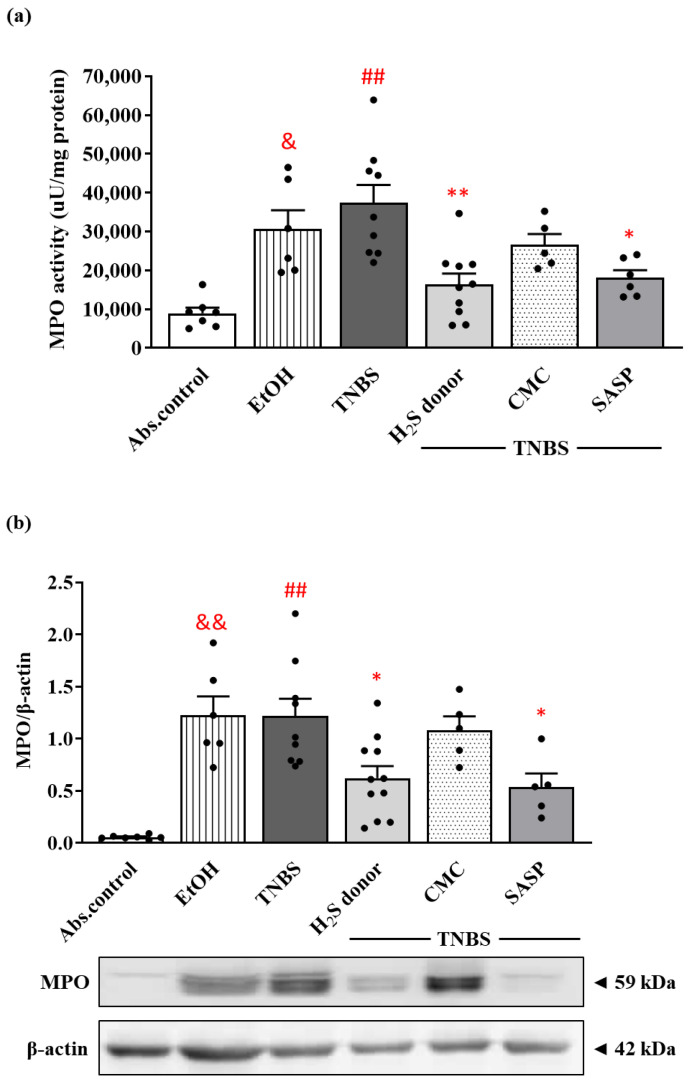
Alterations of the colonic myeloperoxidase (MPO) enzyme activity (**a**) and expression (**b**) following H_2_S donor administration in TNBS-induced rat colitis. Absolute control (no treatment); EtOH (50% ethanol, the solvent of TNBS); TNBS (2,4,6-trinitrobenzenesulfonic acid enema); H_2_S donor (TNBS + 18.75 µM/kg/day Lawesson’s reagent); CMC (TNBS + 0.5% carboxymethylcellulose, the vehicle of Lawesson’s reagent); SASP (TNBS + 50 mg/kg/day sulfasalazine, as a positive control). Results are presented as mean + S.E.M.; *n* = 5–11/group; one-way ANOVA, Holm–Sidak post hoc test, ## *p* < 0.001 absolute control vs. TNBS; * *p* < 0.05, ** *p* < 0.001 TNBS vs. TNBS + treatments; & *p* < 0.05, && *p* < 0.001 absolute control vs. EtOH.

As a marker of inflammation, MPO is released by the activation of neutrophils in inflammatory processes, corresponding to the extent of inflammatory damage. EtOH, the vehicle of TNBS, significantly increased the activity of the enzyme compared to the control group (30,568.63. ± 4866.79 vs. 8958.17 ± 1448.31 µU/mg protein). The induction of colitis by TNBS instillation resulted in an approximately four-fold elevation in MPO activity compared to the control group (35,749.15 ± 4494.80 vs. 8958.17 ± 1448.31 µU/mg protein). Treatment with the protective dose of H_2_S donor significantly diminished the TNBS-increased activity of the enzyme, suggesting a lower infiltration of neutrophils (16,372.51 ± 2794.58 vs. 35,749.15 ± 4494.80 µU/mg protein). Following SASP administration, we also observed a statistically significant reduction in the activity of the colonic MPO compared to the TNBS group (18,059.62 ± 1960.57 vs. 35,749.15 ± 4494.80 µU/mg protein) (Figure 4a).

As Western blot revealed (Figure 4b), MPO was significantly overexpressed following EtOH and TNBS challenge similarly to the activity of the enzyme in both groups compared to healthy controls (1.23 ± 0.18 and 1.22 ± 0.17 vs. 0.06 ± 0.01 relative expression). Release of H_2_S from Lawesson’s reagent caused a significant reduction in the expression of MPO, and equivalent results were seen in the case of the SASP-treated group compared to TNBS (0.62 ± 0.11 and 0.54 ± 0.13 vs. 1.22 ± 0.17 relative expression).

### 3.5. Hydrogen Sulfide Donor Attenuated the Overexpression of HMGB1 by TNBS

**Figure 5:**
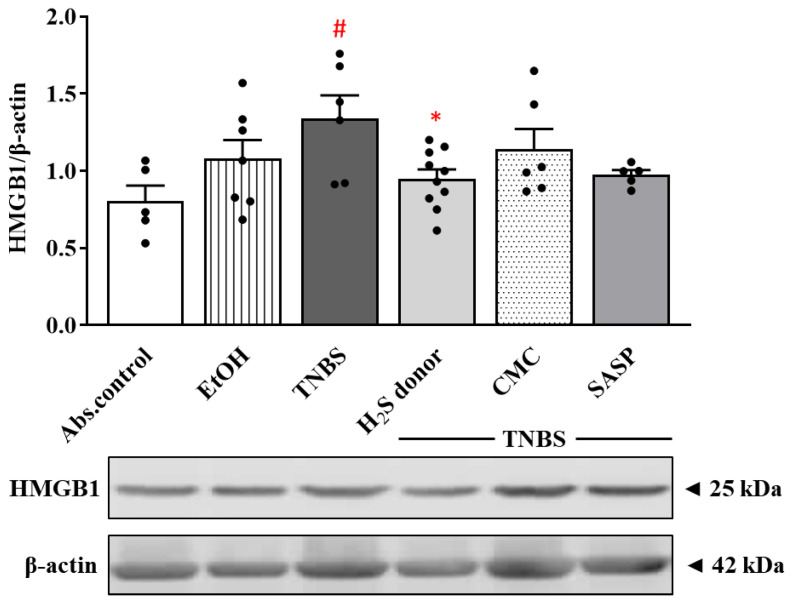
Alterations of the expression of HMGB1 in the treated groups. Absolute control (no treatment); EtOH (50% ethanol, the solvent of TNBS); TNBS (2,4,6-trinitrobenzenesulfonic acid enema); H_2_S donor (TNBS + 18.75 µM/kg/day Lawesson’s reagent); CMC (TNBS + 0.5% carboxymethylcellulose, the vehicle of Lawesson’s reagent); SASP (TNBS + 50 mg/kg/day sulfasalazine, as a positive control). Results are presented as mean + S.E.M.; *n* = 5–10/group; one-way ANOVA, Holm–Sidak post hoc test, # *p* < 0.05 absolute control vs. TNBS; * *p* < 0.05 TNBS vs. TNBS + H_2_S donor treatment.

The expression of HMGB1 was also detected using the Western blot technique. The colonic HMGB1 levels showed a statistically significant increase in the TNBS group compared to the control group (0.80 ± 0.10 vs. 1.34 ± 0.15 relative expression). Treatment with the protective dose of H_2_S donor significantly reduced the expression of HMGB1 compared to the TNBS group (0.95 ± 0.06 vs. 1.34 ± 0.15 relative expression). Although SASP treatment also caused a decrease in HMGB1 levels, there was no significant difference compared to the TNBS group (Figure 5).

### 3.6. Effects of Hydrogen Sulfide Donor on the Expression of NF-kB p65 Subunit in TNBS-Induced Rat Colitis

**Figure 6:**
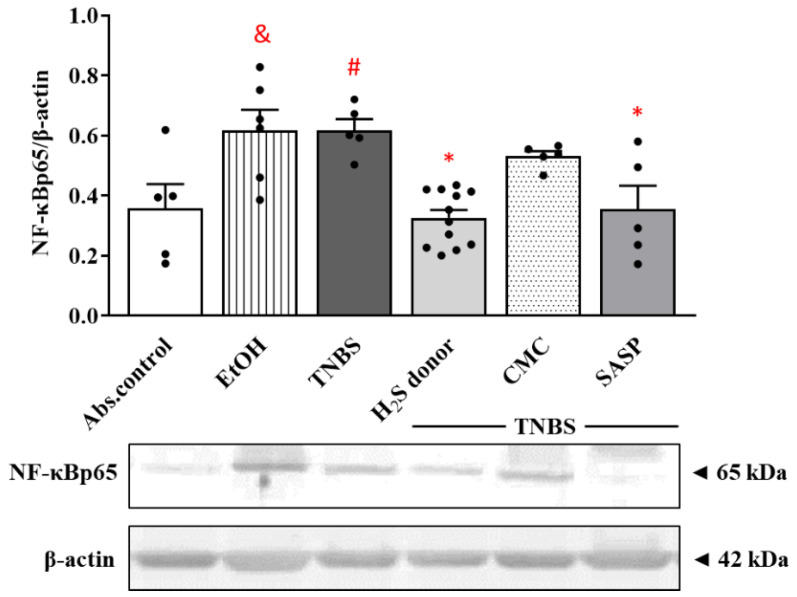
Changes in the expression of NF-κB p65 subunit following TNBS administration and treatment with H_2_S donor in the colon. Absolute control (no treatment); EtOH (50% ethanol, the solvent of TNBS); TNBS (2,4,6-trinitrobenzenesulfonic acid enema); H_2_S donor (TNBS + 18.75 µM/kg/day Lawesson’s reagent); CMC (TNBS + 0.5% carboxymethylcellulose, the vehicle of Lawesson’s reagent); SASP (TNBS + 50 mg/kg/day sulfasalazine, as a positive control). Results are presented as mean + S.E.M.; *n* = 5–12/group; one-way ANOVA, Holm–Sidak post hoc test, # *p* < 0.05 absolute control vs. TNBS; * *p* < 0.05 TNBS vs. TNBS + treatments; & *p* < 0.05 absolute control vs. EtOH.

As a pivotal inflammatory transcription factor, NF-κB has been implicated in the pathogenesis of colitis. As Figure 6 shows, EtOH and TNBS significantly enhanced the expression of NF-κB p65 subunit compared to the absolute control group (0.62 ± 0.07 and 0.62 ± 0.05 vs. 0.36 ± 0.08 relative expression). Release of H_2_S from Lawesson’s reagent resulted in a statistically significant attenuation in the colonic expression of NF-κB p65 subunit compared to TNBS groups (0.33 ± 0.03 vs. 0.62 ± 0.05 relative expression). Orally administered SASP also significantly diminished the level of NF-κB p65 protein compared to the TNBS group (0.35 ± 0.08 vs. 0.62 ± 0.05 relative expression).

### 3.7. Hydrogen Sulfide Donor Administration Elevated the Expression of UCHL-1 Deubiquitinating Enzyme

**Figure 7:**
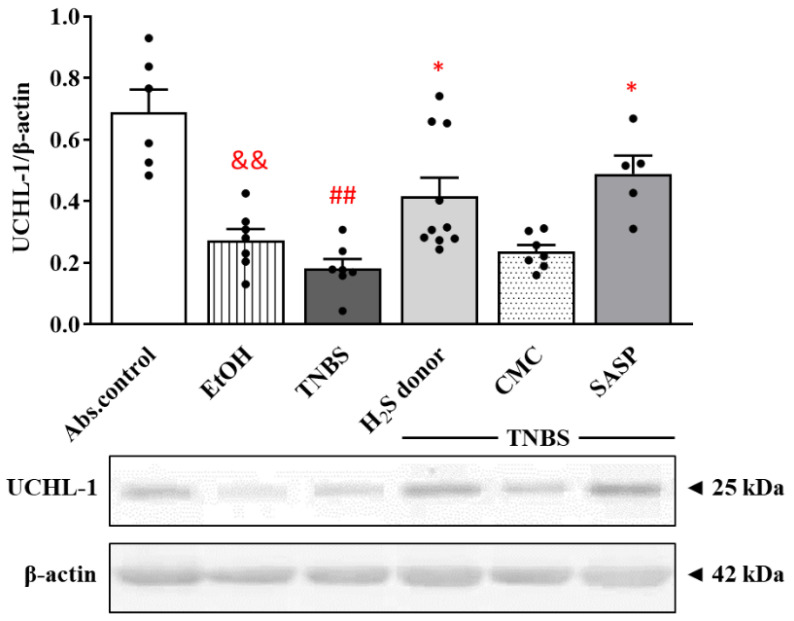
Changes in the colonic expression of UCHL-1 in different treated groups. Absolute control (no treatment); EtOH (50% ethanol, the solvent of TNBS); TNBS (2,4,6-trinitrobenzenesulfonic acid enema); H_2_S donor (TNBS + 18.75 µM/kg/day Lawesson’s reagent); CMC (TNBS + 0.5% carboxymethylcellulose, the vehicle of Lawesson’s reagent); SASP (TNBS + 50 mg/kg/day sulfasalazine, as a positive control). Results are presented as mean + S.E.M.; *n* = 5–10/group; one-way ANOVA, Holm–Sidak post hoc test, ## *p* < 0.001 absolute control vs. TNBS; * *p* < 0.05 TNBS vs. TNBS + treatments; && *p* < 0.001 absolute control vs. EtOH.

Investigating the effects of treatments on the expression of UCHL-1 deubiquitinase enzyme by Western blot analysis revealed that both 50% EtOH and TNBS instillation caused a significant decrease in its expression compared to the non-treated control group (0.27 ± 0.04 and 0.18 ± 0.03 vs. 0.69 ± 0.07 relative expression). However, we detected a significantly enhanced expression of UCHL-1 in the H_2_S donor treated group compared to TNBS (0.42 ± 0.06 vs. 0.18 ± 0.03 relative expression). Similar to Lawesson’s reagent, we observed a significant increase in the positive control (SASP) group compared to TNBS (0.49 ± 0.06 vs. 0.18 ± 0.03 relative expression). Results are presented in Figure 7.

## 4. Discussion

**Figure 8:**
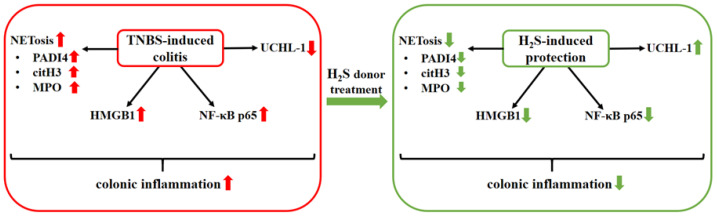
Summary of the presumed protective action of H_2_S donor treatment in experimental rat colitis. 2,4,6-trinitrobenzenesulfonic acid (TNBS); hydrogen sulfide (H_2_S); peptidyl arginine deiminase 4 (PAD4); citrullinated histone H3 (citH3); myeloperoxidase (MPO); high-mobility group box 1 (HMGB1); nuclear transcription factor-kappa B p65 subunit (NF-κB p65); ubiquitin C-terminal hydroxylase L1 (UCHL-1).

Our current study aimed to investigate the effects of H_2_S donor treatment on the expression of NETs-related proteins and inflammatory mediators in an experimentally induced rat model of IBD. We found that the administration of H_2_S donor (18.75 µM/kg/day) attenuated the severity of inflammation in TNBS-induced rat colitis. Our results showed that NETs-associated proteins were significantly increased in colitic colon tissues, while H_2_S donor treatment significantly decreased the expression of these markers. Additionally, the H_2_S donor attenuated the TNBS-provoked expressions of the inflammatory mediators HMGB1 and NF-κB (p65 subunit) and elevated the expression of the UCHL-1 enzyme. These results suggest an inhibitory effect of H_2_S donor in TNBS-colitis through the inhibition of NETs formation, which offers a new potential therapeutic strategy for patients with IBD. The summary of our results is shown in Figure 8.

H_2_S, as an important gasotransmitter, acts as a vasodilator [46], diminishes leukocyte adherence and migration, and, furthermore, reduces edema development [47]. It was demonstrated that H_2_S donor molecules and their drug hybrids might be effective for reducing inflammation, resulting in fewer side effects [48,49,50]. Wallace et al. [51], in their study, revealed that an H_2_S-releasing NSAID, a derivative of naproxen (known as ATB-346), reduced pain and inflammation with less GI injury than naproxen. Moreover, this H_2_S-releasing derivative of naproxen has been tested in human phase2 clinical trial effectively [52]. In our investigation, we used the TNBS-induced rat colitis model, which is a widely accepted model to study IBD [8]. TNBS instillation causes serious transmural inflammation with ulcers and hyperaemia in the colon [9]. In line with previous findings from our laboratory [44], we confirmed in the current study that the administration of H_2_S donor, derived from Lawesson’s reagent, significantly suppressed the severity of inflammation and reduced the extent of ulcers in TNBS-induced rat colitis. The reduction of inflammation was as effective as SASP, a commonly used medical therapy [53]. Chen et al. also reported in dextran sulfate sodium (DSS)-induced colonic inflammation that NaHS treatment decreased inflammation and tissue injury in mice [54]. However, the protective mechanism of H_2_S is still unclear.

Besides phagocytosis and cytokine and ROS production, activated neutrophils can also form and release NETs [25]. IBD is characterized by intense neutrophil infiltration to the site of inflammation [22], and it is increasingly clear that NETs are involved in the pathogenesis of IBD [55,56]. Therrien et al. [57] showed that the recruitment of activated neutrophils in inflamed mucosa is associated with the severity of disease in IBD. Tao Li et al. [56] investigated the presence of NETs formation markers in plasma from patients with IBD. Their results showed significantly elevated plasma levels of DNA and MPO-DNA complexes in active UC or CD patients compared to those with inactive UC or CD and healthy controls. Additionally, an overexpressed citH3 was observed in the colonic tissues of IBD patients. Furthermore, Dinallo et al. [58] provided additional evidence on NETs formation in the inflamed colon of UC patients, demonstrating the co-localization of MPO, NE and citH3 with the DNA by immunofluorescence staining. In our study, we observed the effects of exogenous H_2_S-releasing agent, Lawesson’s reagent, on the expression of NETs-associated proteins in TNBS-induced colitis. Our western blot analysis showed a high expression of PAD4 in colon samples following TNBS instillation. Similarly to PAD4, citH3 also exhibited an increased expression in the TNBS group. We also measured the activity and expression of another NETs-related protein, MPO, which were elevated after the induction of colitis. However, the administration of H_2_S donor significantly decreased the expression of all measured NETs-associated proteins. Our results are consistent with the work of Zhang et al. [59], demonstrating an increased expression of citH3, an elevated gene expression of PAD4 and increased activity of MPO enzyme in TNBS-induced colitis in mice. In their study, the NETs structures were also displayed by immunofluorescence staining and immunohistochemistry. They found that neutrophils formed a web-like structure in the TNBS-treated group, and the treatment with Cl-amidine, a PAD4 inhibitor, led to a reduction in NETs formation. Additionally, Chumanevich et al. measured increased PADI2 and PAD4 levels in DSS-induced colitis in mice [60]. They also demonstrated elevated PAD levels in colon biopsies from UC patients. Furthermore, it was found in animal models that Cl-amidine, a pan-PAD inhibitor, attenuated the severity of several diseases, such as rheumatoid arthritis [61], systemic lupus erythematosus [32], and cancer [62]. In addition, Cl-amidine treatment also reduced NETs formation and suppressed inflammation in DSS- [60] and in TNBS-induced mouse colitis [59]. Furthermore, in a mouse model of lethal lipopolysaccharide (LPS) induced shock, Deng et al. found that treatment with a CitH3 monoclonal antibody attenuated proinflammatory responses and NETs formation [33]. Yang et al. investigated the effects of H_2_S donor on NETosis in diabetic mice [34]. They found that H_2_S donor decreased NETs formation, leading to diabetic wound healing, which is consistent with our current findings in TNBS-induced colitis. These results suggest that the molecules promoting NETs formation could be used as biomarkers for IBD, and H_2_S donor administration may contribute to the attenuation of inflammation and ulcer healing through inhibiting NETosis.

In addition, we examined the expression of the inflammatory mediator HMGB1 in TNBS-induced rat colitis. In line with our previous findings [42], the current results also showed a high expression of HMGB1 in the inflamed colonic tissue. Maeda et al. also detected a colonic overexpression of HMGB1 in the DSS-induced mouse model of UC, while its inhibition by anti-HMGB1 antibody resulted in a decreased extent of inflammation [63]. According to Tadie et al., HMGB1 enhances the NETs formation via toll-like receptor 4 (TLR4), contributing to inflammatory processes and tissue damage [36]. However, in our study, the administration of H_2_S donor markedly attenuated HMGB1 expression. With another H_2_S donor, NaHS, Zhao et al. also demonstrated the inhibitory effect of H_2_S on NETs formation through the reduction of HMGB1 levels in a rat model of hyperhomocysteinemia [35]. These findings, in line with our current results, suggest that the protective effect of H_2_S may rely on the inhibition of HMGB1 expression.

Furthermore, we tested the effects of H_2_S donor administration on the expression of NF-κB. NF-κB is one of the key regulators in inflammatory conditions, and its increased activation is typical in patients suffering from IBD [40]. In the current study, we showed an enhanced expression of NF-κB by TNBS, which was then significantly decreased after treatment with Lawesson’s H_2_S donor. Several studies demonstrated that H_2_S inhibits the degradation of the inhibitor kappa B-α (IκB-α) subunit, which regulates NF-κB activation and translocation [64,65]. Sun et al. showed that citrullination of NF-κB by PAD4 promotes inflammation; thus, the inhibition of this process seems to be a promising therapeutic strategy in inflammation-associated diseases [66]. In our current study, we demonstrated a decreased expression of PAD4 following H_2_S donor administration and an H_2_S-induced NF-κB reduction. Based on these results, we presume that the inhibition of PAD4 expression and the inactivation of NF-κB might contribute to the anti-inflammatory effect of H_2_S. Besides its important role in proteasomal degradation, UCHL-1 as a potential inflammatory mediator is also able to block NF-κB activation by stabilizing IκBα [43]. Here, we demonstrated an attenuated expression of UCHL-1 in rat colitis induced by TNBS, which is consistent with our previous findings [42]. This reduced UCHL-1 expression was significantly restored due to H_2_S donor treatment.

### Limitation

The limitation of our current study is that only one H_2_S donor, namely Lawesson’s reagent, was tested against TNBS-induced colitis to reduce the extent of the damaged area of the colon. However, it would be interesting to check in the future whether other H_2_S donors exert this protection.

Our results showed that NETosis played an important part in the pathogenesis of the TNBS colitis model and H_2_S treatment attenuated the expression of NETs-associated proteins in the colonic tissues. Based on these findings, we concluded that the elevated levels of members of NETs seem to be the consequence of NETosis. The application of immunohistochemical methods may provide morphological evidence of NETosis and may support the protective effects exerted by H_2_S donor in our colitis model, although the limitation of our current study is that we applied only Western blot analysis. NETs formation in the TNBS-induced mouse colitis model was demonstrated by immunohistochemistry; however, it would be worth clarifying the existence of NETosis in the inflamed colonic tissues of rats with the same method.

## 5. Conclusions

In conclusion, we found that exogenous H_2_S decreased inflammation and contributed to ulcer healing by the inhibition of NETs formation and proinflammatory mediators in TNBS-induced rat colitis. Additionally, UCHL-1 seems to contribute to the protective effect of H_2_S. Our findings suggest that NETs formation is involved in the pathogenesis of IBD, and H_2_S, through the inhibition of NETs formation, provides a potential therapeutic option against IBD. Our current result is promising; however, it does not provide comprehensive answers. Therefore, further investigations are needed to reveal the exact role of NETosis in TNBS-induced rat colitis and the protective mechanism of H_2_S in inflammatory conditions.

## Data Availability

All data analysed in this study are included in this published article.
